# An Organoid Model for Translational Cancer Research Recapitulates Histoarchitecture and Molecular Hallmarks of Non-Small-Cell Lung Cancer

**DOI:** 10.3390/cancers17111873

**Published:** 2025-06-03

**Authors:** Camilla T. Ekanger, Maria P. Ramnefjell, Maren S. F. Guttormsen, Joakim Hekland, Kristin Dahl-Michelsen, Maria L. Lotsberg, Ning Lu, Linda E. B. Stuhr, Laurence Hoareau, Pirjo-Riitta Salminen, Fabian Gärtner, Marianne Aanerud, Lars A. Akslen, James B. Lorens, Agnete S. T. Engelsen

**Affiliations:** 1 Centre for Cancer Biomarkers CCBIO, Department of Clinical Medicine, University of Bergen, 5021 Bergen, Norway; camilla.ekanger@uib.no (C.T.E.); maria.ramnefjell@uib.no (M.P.R.); lars.akslen@uib.no (L.A.A.); 2 Centre for Cancer Biomarkers CCBIO, Department of Biomedicine, University of Bergen, 5009 Bergen, Norway; 3 Department of Pathology, Haukeland University Hospital, 5021 Bergen, Norway; 4 Department of Cancer Genomics, Haukeland University Hospital, 5021 Bergen, Norway; 5 Department of Clinical Science, University of Bergen, 5021 Bergen, Norway; 6 Section of Cardiothoracic Surgery, Department of Heart Disease, Haukeland University Hospital, 5021 Bergen, Norway; 7 Department of Thoracic Medicine, Haukeland University Hospital, 5021 Bergen, Norway

**Keywords:** non-small-cell lung cancer (NSCLC), organoids, patient-derived tumor-organoids (PDTOs), histopathology, translational cancer research model development, precision oncology

## Abstract

**Background**: Organoid cultures have received much attention in recent years due to the promise of patient-derived organoid cultures for exploration of personalized cancer treatment strategies. Organoid cultures have been established from a variety of malignancies; however, lack of a thorough histopathological analysis has limited the acceptance of organoid models as translational tools. **Methods**: Here, we aimed to establish patient-derived tumor-organoid (PDTO) models from human non-small-cell lung cancer (NSCLC) resection specimens and provide a thorough histopathological evaluation of the cultures. **Results**: We show that we were able to establish organoid cultures of lung adenocarcinomas (LUADs) and lung squamous cell carcinomas (LUSCs) successfully, and that the organoid cultures of different subtypes of NSCLC preserved the histoarchitecture and growth pattern of the tumors they derive from. Immunohistochemistry and AB-PAS staining confirmed the subtype-specific protein expression pattern and preserved mucin production in LUAD organoids. The genetic abnormalities of the tumors assessed by immunohistochemistry (IHC-P) were preserved in the organoid cultures. **Conclusions**: Our thorough study reveals conserved PDTO histopathology, supports further exploration, and encourages using PDTO models in translational research projects. PDTO models hold remarkable promise as patient-specific models and may be applied to predict therapy response in cases where molecular–pathological analyses pose significant management dilemmas, and they also may provide a platform for exploring the molecular mechanisms of therapy resistance in a biologically relevant model system.

## Simple Summary

Lung cancer is a leading cause of cancer-related morbidity and mortality worldwide. Organoid models are emerging as a translational bridge between basic biomedical science and clinical medicine. In this study, we have successfully established a multicellular 3D model of patient-derived tumor organoids (PDTOs) from non-small-cell lung cancer (NSCLC) tissues, and we have shown, through robust tissue-based analyses, that the histopathological features of the organoid cultures resemble the respective tissues they derive from. The biomimetic organoid models hold great potential to increase our understanding of the molecular mechanisms underpinning disease pathophysiology and therapy resistance, and tumor-organoid models also hold remarkable promise for personalized medicine as patient-specific models and even to predict therapy response in cases where molecular–pathological analyses pose significant management dilemmas.

## 1. Introduction

Lung cancer is a leading cause of cancer-related morbidity and mortality worldwide [1]. Lung cancer has historically been divided into two main types, small-cell lung cancer (SCLC) and non-small-cell lung cancer (NSCLC). In Norway and the United States, NSCLC accounts for approximately 80–85% of lung cancer cases [2,3,4]. NSCLC is further subdivided into two main categories: lung adenocarcinoma (LUAD) and lung squamous cell carcinoma (LUSC). While temporal and geographical distribution of lung cancer incidence varies across the main subtypes, worldwide, for women, LUAD has recently been determined to be the most frequent type of lung cancer in all 185 countries evaluated, and for men, in 150 (81%) of 185 countries [1,5]. Other minor subtypes are large-cell lung carcinoma (LCLC), adenosquamous carcinoma, and sarcomatoid carcinoma [4,6]. The specific cellular origin of the subtypes is largely unknown; however, while LUAD usually develops in more distal locations [7], LUSC usually arises in the epithelial layer of bronchial airways in a sequence from squamous cell metaplasia, via squamous cell dysplasia, to carcinoma. LUAD can further be subdivided according to the major growth pattern displayed: lepidic (growth along pre-existing alveolar structures), papillary (forming papillary structures with fibrovascular cores), acinar (forming glandular structures), solid (dense groups of epithelial tumor cells), and micropapillary (forming small papillary structures lacking fibrovascular cores). The growth patterns have been associated with differences in cancer-specific survival [8,9,10].

In a country like Norway, with public health care and a clear goal of equal access to treatment, treatment of lung cancer depends primarily on the subtype and the disease stage, in addition to age and performance status of the patient [11]. With major advances in molecular testing and the introduction of targeted therapies for LUADs in particular, the distinction between LUAD and LUSC has become increasingly important. Molecular testing is usually performed on pre-operative biopsies when available. In addition to histological evaluation on H&E-stained sections, dual immunohistochemistry (IHC-P) is applied when necessary. Dual IHC-P is preferred over single IHC-P protocols in order to preserve tissue for molecular testing. Thyroid transcription factor-1 (TTF1) is a sensitive marker for pulmonary and thyroid adenocarcinomas. Napsin A is a protease of the pepsin family A, which is essential for the maturation of pro-surfactant protein B (pro-SP-B) and pro-surfactant protein C (pro-SP-C) in type II pneumocytes. Positive TTF-1/Napsin A staining is expected in LUAD. Approved markers for squamous differentiation are p40 and cytokeratin 5/6 (CK5/6) [12].

The current multimodal treatments for lung cancer include surgery, radiation therapy, chemotherapy, and targeted therapies, including immunotherapy, either alone or in combination [13]. Specific therapies are offered to the patients depending on the histologic diagnosis and molecular status of the tumor. Targeted therapies offered to LUAD patients with tumors harboring specific molecular aberrations include drugs against anaplastic lymphoma kinase (ALK) and inhibitors for epidermal growth factor receptor (EGFR) and ROS proto-oncogene 1 (ROS1) [14]. With the development of molecularly targeted drugs, including immune checkpoint inhibitors (ICI), in particular, drugs targeting PD1 and its ligand PD-L1, the treatment of lung cancer has undergone dramatic changes, and the introduction of ICI represents a significant advancement [15]. Nonetheless, only a minority of the patients respond, and the overall disease prognosis for patients with advanced disease remains poor [15,16,17,18,19]. To improve the management of NSCLC, new predictive biomarkers are needed to enable the physician to tailor the multimodal treatment regimens to the individual patient.

Currently available NSCLC models, such as cell lines established from human NSCLC tumors and grown as monolayers (2D), are widely used in preclinical research due to their availability, ease of use, and reproducibility in experimental setups. However, cell line monocultures are not representative of the complex tumor microenvironment and the intricate features of NSCLC. The use of heterotypic spheres consisting of human NSCLC cell lines and human fibroblasts grown as spheres allows the study of tumor cell and stromal cell interactions in vitro [20]. In addition, a range of chemically induced animal models, xenograft models, and genetically modified mouse models of lung cancer are available. Depending on the research question, the various animal models recapitulate particular hallmarks of the disease, and the use of these models has contributed to the understanding of lung cancer carcinogenesis and pathophysiology. The search for complex and representative preclinical human models for the development of effective therapeutic strategies and exploring robust personalized clinical decision-making has led to the development of the organoid models. The Clevers’ laboratory [21] has pioneered the development of physiologically relevant 3D in vitro models, namely organoids, derived from induced pluripotent stem cells or adult stem cells derived from patient-specific material, the latter referred to as patient-derived organoids (PDOs). Patient-derived tumor-organoids (PDTOs) are generated from the malignant cells capable of initiating tumor organoid cultures.

The PDTO models have proven to be a powerful tool in numerous biomedical studies, especially in their application to the advancement of personalized medicine [22,23]. Drug sensitivity of cancer cells cultured on 2D plastic surfaces has been shown to differ dramatically from the sensitivity of cells in a 3D culture system [24]. In terms of lung cancer, PDTO models derived from NSCLC resection specimens have been used to assess how tumors may respond to various treatments by utilizing genetic and molecular characteristics of the patient-specific tumor [25,26]. Besides the apparent goal of improving the availability of relevant and representative human preclinical models of NSCLC subtypes for research purposes, an additional goal is to apply PDTO cultures for predictive testing of drug response in the clinic to allow personalized medicine based on functional testing of the patients own PDTO culture, with the PDTO cultures serving as the patients avatars [27]. When pre-operative tumor material for traditional predictive testing is scarce, it would also be an advantage if PDTO cultures could be applied to amplify the tumor material available for testing to guide clinical decision-making. In addition to our own work, recent studies have shown that a main challenge caused by an overgrowth of normal epithelial cells in the culture remains [28,29]. It is crucial to ensure that the patient-derived tumor specimen and the corresponding PDTO cultures are comparable with respect to histological and molecular characteristics and growth patterns. The use of PDTO models is discouraged unless precautions are taken to prevent normal epithelial cell overgrowth [29]. Therefore, implementing PDTO into clinical practice requires improvements in the current protocols for generating PDTO models.

The aim of this study was to establish and evaluate NSCLC PDTO models and provide a thorough characterization of the histoarchitecture and growth pattern of the tumor organoids compared to the malignant tumors they derive from. We believe that the comprehensive characterization of PDTO culture and the histopathological characterization described here will be of value for future studies seeking to improve the PDTO models, with an ultimate aim of increasing the application of these biologically relevant tools in translational research projects and co-clinical trials.

## 2. Materials and Methods

### 2.1. Human Tissue Specimens for Organoid Model

NSCLC tumor specimens were obtained from elective lobectomies performed at Haukeland University Hospital (HUH), Bergen, Norway. Prior to the collection of surgical resection specimens, written informed consent was obtained in line with the requirements from the regional ethical committee (REK approval number #66610). One tumor sample per patient (*n* = 11 patients) was collected for this study. Patients eligible for enrollment in the study were identified in multidisciplinary Thorax-meetings at Haukeland University Hospital. Great care was taken to ensure that the allocation of tumor tissue for research did not interfere with the regular diagnostic procedures (e.g., distance to pleura, invasion, tumor measurements, etc.). In order to avoid any potential impact on diagnostics where the distance to pleura could affect downstream procedures, all tumors with suspected infiltration of pleura were excluded. In order to comply with the ethical standards, the only deviation from the standard procedure is that the excised tumor specimen is collected at the operating theater and immediately transported in a cooled container to the Department of Pathology, where a tumor specimen for organoid culture was carefully excised by an experienced pathologist (M.R.). The remaining resection specimens underwent standardized pathological analyses according to the institutional guidelines. Excised tissue specimens for PDTO culture were placed in organoid washing medium, AdDF+++ (Table 1); transported on ice to a biosafety level 2 laboratory; and processed within 30 min, according to the protocol described below.

### 2.2. Tissue Processing for Organoid Culture

The method developed by Sachs and colleagues [30], with modifications described by Hoareau and colleagues [31], as well as Ekanger and colleagues [32,33], for normal airway and lung organoids was used as the foundation for establishing PDTO cultures in this study. Briefly, the fresh tumor tissue was finely minced in a sterilized glass Petri dish, using a pair of surgical scalpels. The minced tissue was transferred into a 50 mL falcon tube containing AdDF+++ (Table 1). To digest the tissue, 2 mg/mL collagenase (C9407, Sigma-Aldrich, Saint Louis, MO, USA) was added. The falcon tube was then placed on an orbital shaker and subjected to 200 rpm at 37 °C for a duration of 2 h. Following digestion, any remaining tissue was mechanically sheared using a sterilized 10 mL glass Pasteur pipette, and the resulting mixture was filtered through a prewet 100 μm cell strainer (Corning, New York, NY, USA, 431752). The pellet was resuspended in 10 mL of AdDF+++ (Table 1) prior to an additional centrifugation step. To lyse residual erythrocytes, 2 mL of red blood cell lysis buffer (11814389001, Roche, Basel, Switzerland) was added to the pellet and incubated for 5 min at room temperature (RT). Next, 10 mL AdDF+++ (Table 1) was added, followed by collection of cells with a final centrifugation at 400× *g* for 5 min.

### 2.3. Organoid Expansion and Differentiation

The digested lung cancer tissue was placed on ice and resuspended in ice-cold Growth Factor Reduced Matrigel (356231, Corning, New York, NY, USA). Subsequently, approximately 12,500 freshly isolated epithelial cells were resuspended per 50 μL droplet of Matrigel, and the Matrigel–cell mixture was seeded per well of pre-warmed (37 °C) 24-well plates (83.3922, SARSTEDT, Nümbrecht, Germany). The plates were inverted and maintained at 37 °C for 30 min to allow for solidification of the Matrigel domes, while preventing organoids from settling at the bottom of the dome, near the plastic surface. Following solidification of the Matrigel domes, 500 μL of airway organoid alveolar (AO-A) differentiation medium or airway organoid bronchiolar (AO-B) differentiation medium (Table 2) was gently added to each of the dome-containing wells of the 24-well plate. Only in the case of AO-A medium, **CHIR99021 was added, as described [31]. PBS was added to the empty wells on the edge of the plate to prevent excessive evaporation. The plates were kept in a humidified 5% CO_2_ and 5% O_2_ multigas incubator set at 37 °C [34,35]. To ensure an optimal growth environment, the medium was changed every 4 days, and the organoids were passaged when confluent, approximately every two weeks. Organoids from the various donors were anonymized and referred to with the sample ID, L1-L11.

### 2.4. Passaging of Organoids

Passaging of organoids was performed every 2–3 weeks, depending on the growth rate and the confluence of the culture. To dissolve Matrigel, 1 mL of ice-cold AdDF+++ (Table 1) was added to each well, and the organoid–medium mixture was vigorously pipetted up and down to dissolve prior to collection and transfer to a falcon tube. An additional 1 mL of ice-cold organoid washing medium was added to the same well in an attempt to harvest all cells. The organoids were centrifuged at 400× *g* for 5 min at 20 °C. After centrifugation, the pellet was resuspended in 2 mL TrypLE (12604013, Gibco, Taastrup, Denmark) and incubated at 37 °C for 5 min to enable organoid dissociation into single cells. To inactivate TryPLE, an additional 10 mL of AdDF+++ (Table 1) was added, and the organoid suspension was centrifuged at 400× *g* for 5 min at 20 °C. The cell pellet was resuspended in cold Growth Factor Reduced Matrigel (356231, Corning, USA) and distributed in a 1:5–1:6 ratio onto pre-warmed 24-well plates (83.3922, SARSTEDT, Nümbrecht, Germany). After the transfer, the organoid medium was supplemented with the ROCK inhibitor, Y-27632 (72304, StemCell Technologies, Cambridge, MA, USA), for a duration of 4 days. Afterward, the Y-27632 was removed from the organoid medium.

### 2.5. Cryopreservation of Organoids

To cryopreserve the organoids, Matrigel domes were initially dissolved by adding 1 mL of ice-cold AdDF+++ (Table 1). To ensure complete organoid harvest, an additional 1 mL of ice-cold AdDF+++ was added. The organoids were centrifuged at 400× *g* for 5 min at 20 °C, followed by TrypLE treatment for 5 min at 37 °C. AdDF+++ was used to inactivate the TryPLE, and the organoid suspension was centrifuged at 400× *g* for 5 min at 20 °C. The pellet was resuspended in a freezing solution (BBD01, Bambanker Direct, Düren, Germany). Subsequently, 500 μL of the resuspended mixture was transferred into a cryotube. To ensure controlled cooling, the cryotubes were placed within a CoolCell LX freezing container (CLS432002, Corning, New York, NY, USA). The cryotubes were stored at −80 °C for 24 h before being transferred to liquid nitrogen for long-term storage.

### 2.6. Formalin Fixation and Paraffin Embedding (FFPE) of Organoids

To collect the organoids, medium was removed from the wells, and the Matrigel domes were dissolved by adding ice-cold AdDF+++ (Table 1). The organoids were transferred to a 15 mL falcon tube, followed by centrifugation at 400× *g* for 5 min at RT. The supernatant was discarded before adding 10 mL fresh AdDF+++ to the falcon tube, followed by a new centrifugation at 400× *g* for 5 min at RT. The organoid pellet was washed once with PBS and fixed at RT for 24 h, using 3.7% formaldehyde (252549, Sigma-Aldrich, St. Louis, MO, USA). Following the fixation process, the organoids were collected by centrifugation at 400× *g* for 5 min and washed with PBS twice. Subsequently, the organoids were processed for FFPE as previously described [32].

### 2.7. H&E Staining

The H&E staining to assess histology was carried out according to established protocols by the Molecular Imaging Centre, as previously described by Feldman and Wolfe [36]. The H&E-stained samples were scanned using the Olympus VS120 S6 Slide Scanner (Olympus, Tokyo, Japan), and the images were captured using the pike F-505 color camera (Allied Vision, Stadtroda, Germany). ImageJ software (version 1.53) was used to incorporate scale bars.

### 2.8. Alcian Blue–Periodic Acid–Schiff (AB-PAS) Staining

To evaluate the presence of mucin, alcian blue–periodic acid–Schiff (AB-PAS) staining was performed according to standard departmental protocols, using Tissue-Tek Prisma (Sakura Fintek, Torrance, CA, USA). The process involved staining the organoids with an alcian blue solution at pH 2.5 for 8 min, applying periodic acid for 10 min, and subsequently rinsing for 3 min after each staining step. Following this, the organoids underwent staining with Schiff reagent for 15 min, a 5 min tap-water rinse, and a 30 s hematoxylin stain. Xylene was used to clear the sections for 2 min. Subsequently, the AB-PAS-stained samples were scanned with a 40× objective, using the VS120 S6 Slide scanner (Olympus, Tokyo, Japan), and the Pike F-505 color camera (Allied vision, Stadtroda, Germany) was employed for image capture. The ImageJ software (version 1.53) was used to add scale bars.

### 2.9. Immunohistochemistry-Paraffin (IHC-P)

Immunohistochemical staining of paraffin sections (IHC-P) was performed on tumors and organoids to verify the histological subtype. IHC-P dual staining of TTF1/Napsin A and p40/CK5/6 was performed at the Department of Pathology at Haukeland University Hospital. IHC-P was performed using the Benchmark Ultra Autostainer system (Roche) and stained with antibodies for thyroid transcription factor 1 (TTF1) (NCL-L- TTF1-1, Leica), Napsin A (352M-96, Cell Marque, Rocklin, CA, USA), p40 (ACI 3066C, Biocare Medical, Pacheco, CA, USA), and cytokeratin 5/6 (CK5/6) (M7237, Dako Agilent Santa Clara, CA, USA). Detection kits used were UltraView Universal DAB detection kit (760–500, Roche) and UltraView Universal Alkaline Phosphatase Red Detection kit (760–501, Roche). PD-L1 expression of cancer cells was performed using the Ventana SP263 kit or the Dako 22C3 kit, as specified in the Results section for each case.

Detection of ALK (D5F3) and ROS proto-oncogene 1 (ROS1) rearrangements was determined by IHC-P, according to the routine for clinical specimens. No cases were positive in our cohort; if found positive by IHC-P, the cases would have been verified by FISH or NGS. Depending on the clinical setting, additional markers were examined (e.g., CK7, CKAE1/SE3, PSA, PSMA, and CK20), according to the institutional procedures. Stained FFPE specimens were scanned using the VS120 S6 Slide Scanner (Olympus, Tokyo, Japan), equipped with a 20× and 40× objective.

Pathologists’ TNM scoring of the surgical specimens was performed according to the eighth edition of the UICC classification [37].

### 2.10. Molecular–Pathological Analyses of Tissue from Clinical Routine FFPE Specimens and Organoid Tissues

Molecular–pathological analyses to determine targetable genetic aberrations were performed using tissue from the pre-operative biopsy specimens for all LUAD samples, apart from sample L9, where tissue from the surgical specimen was used for analysis. Molecular–pathological analyses were performed as per institutional procedures (Department of Pathology, Haukeland University Hospital and Department of Cancer Genomics, Haukeland University Hospital).

All but one of the adenocarcinomas underwent a complete molecular pathological evaluation to determine potential molecular targets. The molecular pathological analysis included ALK expression evaluated by IHC-P, ROS by IHC-P (as described above). EGFR, PIK3CA, BRAF, NRAS, and KRAS mutation analyses were performed by NGS targeted sequencing, using NGS TruSight Tumor 15 (Illumina, 20005610) or NGS Ampliseq for Illumina Focus panel (Illumina, 20019164) using the MiniSeq System (Illumina), as per manufacturers recommendations. Both NGS TruSight Tumor 15 (Illumina, San Diego, CA, USA) and NGS Ampliseq Focus panels (Illumina) were determined to have a lower detection limit of 5–10% tumor cells. E.N.Z.A Tissue DNA kit (Omega Bio-tek, Norcross, GA, USA, D3396-01) were used for DNA purification of FFPE tissues. The bioinformatics software Alissa Interpret v5.2 (Agilent Technologies, Santa Clara, CA, USA) was used for NGS data analyses.

### 2.11. Genomic DNA Extraction of PDTO Cultures for Sanger Sequencing/ddPCR

Prior to the start of the protocol, the organoids were dissolved into single cells, as described above; washed with cold PBS; centrifuged for 5 min at 400× *g*; and resuspended in 200 mL PBS. The E.Z.N.A kit was used for genomic DNA extraction (D3396-02, Omega Bio-Tek, Norcross, GA, USA). Briefly, 20 mL of proteinase K (1114886, Qiagen, Venlo, The Netherlands) and 4 mL of RNAse were added for protease digestion and to degrade RNA, respectively, followed by the addition of 220 mL BL buffer and a 10 min incubation on a shaking heat-block at 55 °C. Next, 220 mL 100% ethanol was added prior to transfer of the sample to a HiBind DNA mini column inserted in a collection tube. All centrifugations were performed at 16,000× *g*, and filtrate was discarded following centrifugation. The collection tube with the inserted column was centrifuged for 1 min. This was followed by the addition of 500 mL of HBC buffer and a 30 s centrifugation. The DNA mini-column was inserted into a new collection tube, and 700 mL of DNA wash buffer was added, followed by centrifugation for 30 s. This step was performed twice. To dry the DNA mini-column, the empty column was centrifuged for 2 min, before it was transferred to a nuclease-free 1.5 mL Eppendorf tube. The elution buffer was heated to 70 °C before 100–200 mL was added to the column and incubated at 2 min at RT, followed by a 1 min centrifugation. This step was performed twice. The nanodrop was used to measure the final concentration of nucleic acid in the sample. Eluted DNA was stored at 4 °C until further processing. For L8 and L11, we aimed to verify the detected KRAS variants of clinical significance found in the clinical samples of DNA extracted from organoid cultures L8 and L11, utilizing Sanger Sequencing for KRAS exon 2. When KRAS was not detected by Sanger Sequencing, requiring 25–30% tumor cells, we tried a real-time PCR, requiring only 10% tumor cells. Thyroid Cancer Mutation Detection Kit (THDNA-RT64, EntroGen, Woodland Hills, CA, USA) was used, using the ABI Applied Biosystems Fast 7500 Real-Time PCR system (ThermoFisher, Waltham, MA, USA). In L8, KRAS variant of clinical significance was not detected, and in L11, G12C/c.34G>T was detected, but outside the reference area used for the clinical samples.

## 3. Results

### 3.1. The Feasibility and Flexibility of the Model

The protocol developed by Sachs and colleagues [30], with modifications [31,32], was used as the basis for the establishment of NSCLC PDTO cultures in this study. We established organoids from a total of 11 NSCLC tumor resection specimens. The protocol demonstrated a 100% success rate in the establishment of organoid cultures from the NSCLC resection specimens. Furthermore, we did not observe contamination in the form of bacterial or fungal infections of the cultures.

**Figure 1:**
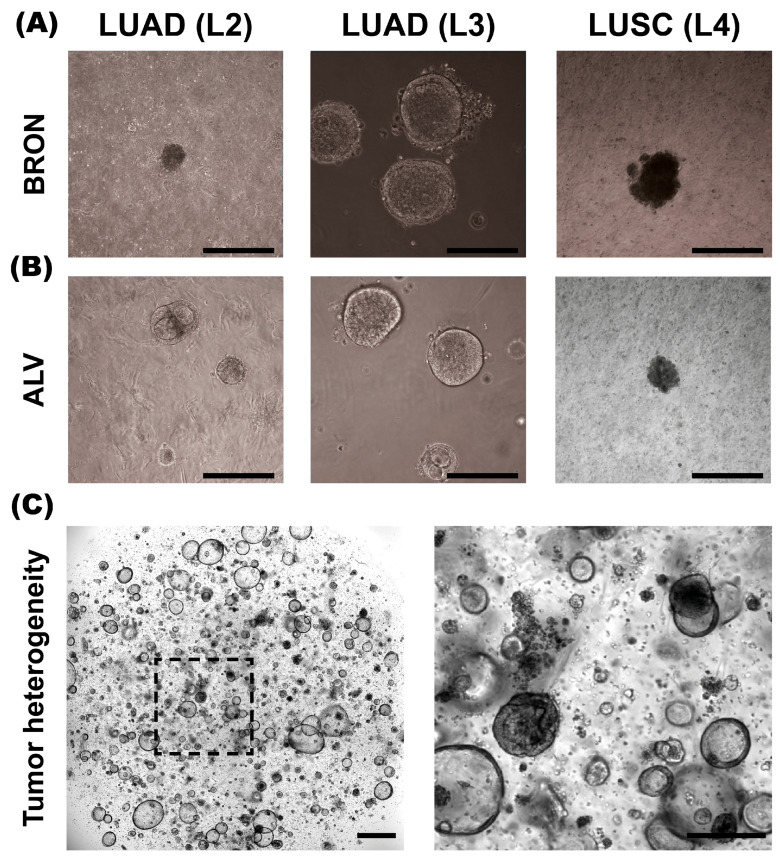
Morphology of BRON and ALV differentiated patient-derived tumor organoids (PDTOs) in culture. (**A**) Representative brightfield images of PDTO cultures L2, L3, and L4 established in bronchiolar (BRON) differentiation culture media. (**B**) Representative brightfield images of PDTO cultures derived from L2, L3, and L4, cultured in alveolar (ALV) differentiation culture medium. (**C**) Representative image of L11-derived PDTO/BRON culture obtained by IncuCyte Live Cell analysis system (Sartorius). Scale bars equal 300 μm for all images, except (**C**), left image (800 μm).

Establishment of tumor organoids cultured in alveolar and bronchiolar differentiation mediums was evaluated for specimens L2-L4 (Figure 1). We found that organoid culture in both media was successful. Except for L2, enhanced organoid growth was observed in the bronchiolar differentiation medium compared to the alveolar differentiation medium. Consequently, subsequent specimens (L5-L11) were cultured only in the bronchiolar differentiation medium.

A subset of the PDTO cultures were successfully cultured for up to seven passages, or around 14 weeks in total. Yet, the majority of the cultures showed a limited capacity for long-term expansion. For instance, L2 cultured in the bronchiolar medium was successfully established. However, the organoids stopped proliferating after the first passage. Furthermore, following the cryopreservation of PDTO cultures, re-establishing the cultures proved to be challenging for some specimens, with a small proportion failing to re-establish. In contrast, the re-culturing process for normal lung organoids proved to be more successful following cryopreservation [31,32,33].

Table 3 gives an overview of the patients whose tumor resection specimen tissue was included in this study. The table gives an overview of the sample ID, assigned gender (M = male/ F = female), NSCLC subtype, and pTNM status.

### 3.2. The Organoid Tissues Preserve Key Histological Hallmarks of the Corresponding Patient Lung Adenocarcinomas (LUADs)

The main aim of this study was to explore whether the histological hallmarks of the tumor specimens were recapitulated in the corresponding PDTO cultures. In general, we found that, for LUADs, the histoarchitecture and growth pattern determined by a pathologist’s evaluation of the histological patient specimens were conserved in the organoid tissues.

#### 3.2.1. L1

**Figure 2:**
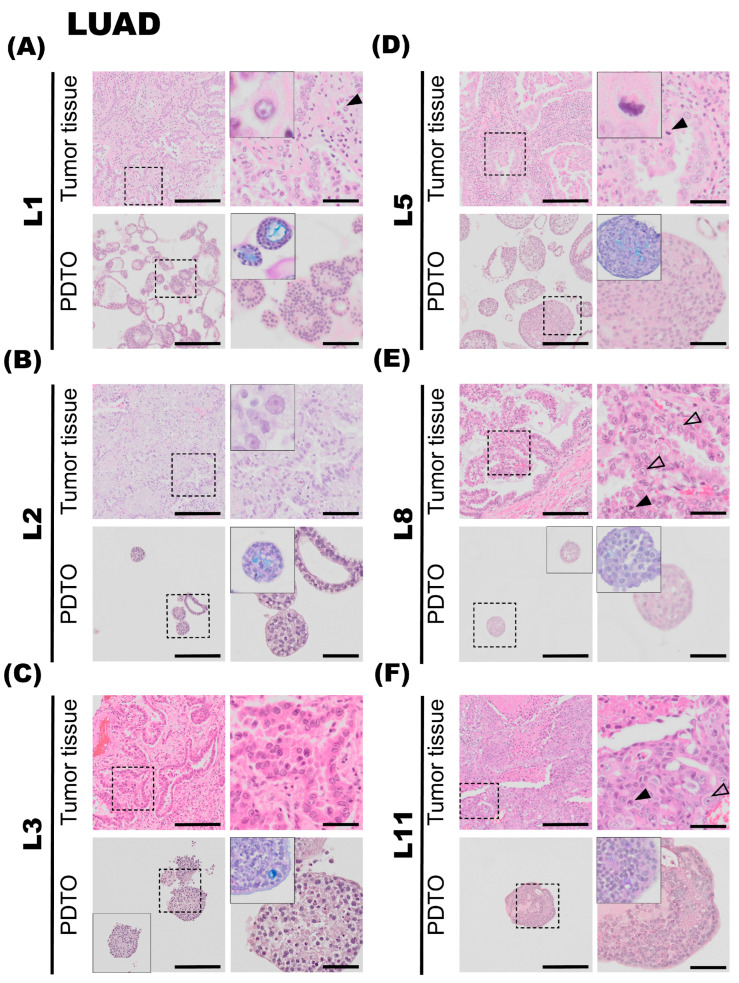
Histology of lung adenocarcinoma (LUAD) tumor specimens and the corresponding patient-derived tumor organoid (PDTO) cultures. Histology of LUAD tumor specimens (upper rows) and the corresponding PDTO cultures from bronchiolar differentiation medium (lower rows) were assessed on hematoxylin and eosin (H&E)-stained formalin-fixation and paraffin-embedding (FFPE) sections. AB-PAS staining of the PDTO cultures is shown in the zoomed inserts. (**A**) Tumor specimen L1 (upper panel) is a well-differentiated LUAD characterized by predominantly acinar and partly lepidic growth pattern. Prominent nucleoli (black arrowhead) are observed. A majority of L1 PDTOs (lower panel) grow as hollow organoids, with multiple layers of cells lining the outer rim and moderate nuclear atypia. (**B**) Tumor specimen L2 (upper panel) is a LUAD with a predominant lepidic growth pattern and moderate nuclear atypia. L2 PDTO (lower panel) consists of a mix of compact and hollow organoids. (**C**) Tumor specimen L3 (upper panel) is a LUAD with a predominant acinar growth pattern and prominent nucleoli. L3 PDTO culture (lower panel) grow compact organoids. (**D**) Tumor specimen L5 (upper panel) is a LUAD characterized by an acinar growth pattern. Mitotic figures are readily observed (black arrowhead). L5 PDTO culture (lower panel) consists of compact structures. (**E**) Tumor specimen L8 (upper panel) is a LUAD with a predominant papillary growth pattern. Cells with prominent nucleoli (transparent arrowheads) and mitotic figures (black arrowhead) are observed. L8 PDTO culture (lower panel) consists of compact organoids with moderate nuclear atypia. (**F**) Tumor specimen L11 (upper panel) is a LUAD characterized by solid growth with only scattered acinar structures. Mitotic figures (black arrowhead) and prominent nucleoli (transparent arrowhead) are observed. L11 PDTO culture (lower panel) grows as compact organoids. Scale bars equal 200 μm (left) and 50 μm (right, zoomed inserts).

Tumor specimen L1 is a well-differentiated LUAD characterized by a predominant acinar and partly lepidic growth pattern. The epithelial tumor cells show moderate-to-prominent nuclear atypia and a high nucleus-to-cytoplasm ratio. The nuclei are pleomorphic with condensed chromatin and partially vesicular, some with prominent nucleoli (Figure 2A, upper panel, black arrowhead). Intracellular mucin vacuoles are only seen in a few of the epithelial tumor cells. PDTOs derived from L1 display mainly circular/oval structural morphology, resembling PDOs derived from normal adult stem cells [32], but with less differentiation and more atypia. Most of the organoids present a central lumen with multiple layers of cells lining the outer rim of the organoid structures, as seen in Figure 2A, lower panel. The organoids were generally around 100–200 μm in diameter. The PDTO culture showed moderate nuclear atypia. Furthermore, the AB-PAS histochemistry, used to detect mucin production, revealed that the organoids produce mucin and that most hollow lumens present in the PDTO tissues were positive for mucin (Figure 2A, lower panel).

#### 3.2.2. L2

The second lung tumor specimen (L2) is an adenocarcinoma characterized by a predominant lepidic growth pattern, with smaller areas of acinar growth, as seen in Figure 2B, upper panel. The epithelial tumor cells show moderate nuclear atypia and intra-cytoplasmatic vacuoles are present. Low cell-division rates are typically observed in the lepidic adenocarcinomas, and the same observation applies to this specimen. The NGS results for the L2 tumor specimen were negative for all tested mutations, and PD-L1 IHC (clone 22C3) was negative. The PDTOs derived from this tumor were difficult to culture in the bronchiolar medium over a prolonged period of time; in fact, we were unable to expand the bronchiolar differentiated organoids beyond the first passage. At a histological level, the PDTOs derived from L2 displayed largely similar characteristics compared to the tumor. PDTOs from tumor L2 grew as a mix of compact and hollow organoids. Few mitotic figures were observed on the H&E-stained sections of L2 PDTOs. The organoids were typically 50–150 μm in diameter. Subsequently, the AB-PAS stain shows that the organoids have moderate mucin production.

#### 3.2.3. L3

Tumor specimen L3 is a LUAD characterized by an acinar growth pattern, displaying similar architectural and nuclear atypia as L1. The L3 PDTO culture is quite heterogeneous and grows as compact organoid structures. The culture proliferated well in both alveolar and bronchiolar differentiation medium, and the average organoids were 100–250 μm in diameter. The AB-PAS stain revealed that these organoids efficiently produced mucin. H&E-stained sections of the tumor specimen compared to the derived organoids show comparable histological characteristics and growth patterns, with moderate nuclear pleomorphism, with some nuclei exhibiting enlargement and prominent nucleoli, as seen in Figure 2C. The NGS results of the patient tumor specimen revealed that L3 is positive for EGFR deletion in chromosome 19. EGFR deletion in chromosome 19 was confirmed in organoids by EGFR del19 immunofluorescent staining. PD-L1 IHC (clone 22C3) was negative.

#### 3.2.4. L5

The fifth lung tumor specimen (L5) is a LUAD largely characterized by an acinar growth pattern, as seen in Figure 2D, upper panel. Furthermore, the tumor also consists of areas of papillary growth, as well as solid isolated groups consisting of only a few malignant cells. Some infiltrating single epithelial tumor cells are observed. The specimen displays a high degree of nuclear pleomorphism: the nuclei show large variations in size, shape, and chromatin structure. Additionally, the tumor specimen consists of some areas of necrosis, and mitotic figures are readily observed in the specimen (Figure 2D, upper panel, black arrowhead). The organoids derived from tumor specimen L5 display histological characteristics comparable to the tumor specimen. Organoids derived from L5 mostly consist of compact structures (Figure 2D, lower panel). The organoids are usually 50–150 μm in diameter. The AB-PAS stain of the organoids shows some mucin production with prominent intracytoplasmic mucin vacuoles (Figure 2D, lower panel). The NGS results from the patient tumor were negative for all tested genetic variants. PD-L1 IHC (clone 22C3) was negative.

#### 3.2.5. L8

Tumor specimen L8 primarily shows a papillary growth pattern. However, the tumor is heterogeneous, and areas of acinar structures, lepidic, and solid growth are detected. Areas showing acinar and papillary growth patterns are shown in Figure 2E, upper panel. The epithelial tumor cells have an intermediate amount of eosinophilic cytoplasm, and some cells have an intra-cytoplasmatic mucus-containing vacuole. The shape of the nuclei is round to oval, with nuclear indentations and condensed chromatin, consistent with moderate nuclear atypia. Some cells have prominent nucleoli (Figure 2E, upper panel, transparent arrowheads). Mitotic figures are easily found (Figure 2E, upper panel, black arrowhead). The molecular analysis showed that the tumor had a mutation in KRAS (G12C). PD-L1 ICH (clone 22C3) was negative. The organoids derived from tumor specimen L8 were typically 80–140 μm in diameter, consisting of a mixture of compact and hollow structures. H&E-stained sections of the organoids show comparable histological characteristics and growth patterns, with moderate eosinic cytoplasm and moderate nuclear atypia, some showing small nucleoli. AB-PAS stain showed some intracytoplasmic mucin-containing vacuoles in the specimen.

#### 3.2.6. L11

The tumor tissue of specimen L11 consists of solid growth, with only scattered acinar structures. The epithelial tumor cells were moderately enlarged with hyperchromatic nuclei of irregular shapes and some with prominent nucleoli (Figure 2F, upper panel, transparent arrowheads). Some mitotic figures are seen (Figure 2F, upper panel, black arrowhead), as well as necrotic areas. Immunohistochemistry showed that the tumor cells were positive for TTF1 and Napsin A and negative for p40 and CK5/6. Taken together, the tumor morphology and the immune profile are consistent with LUAD. PD-L1 IHC (clone SP263, Ventana, Chicago, IL, USA) showed cell membrane staining in 20% of viable epithelial tumor cells, giving a tumor percentage score (TPS) of 20%. IHC-P of ALK (D5F3) and ROS1 were negative. Organoids derived from tumor specimen L11 show moderate-to-sparse nuclear pleomorphism, some with prominent macronucleoli, defined as nucleoli of the same size or larger than a red blood cell. The aggressive growth pattern observed in the tumor specimen can possibly explain the efficient expansion of PDTO cultures derived from this specimen.

### 3.3. Histological Traits of Lung Squamous Cell Carcinomas (LUSC) Are Well Preserved in Organoid Cultures

In general, the LUSC tumors have very compact growth, which is also a prominent feature reflected in organoid cultures. The LUSC PDTO cultures appear very dense under the light microscope and are readily distinguished from LUAD PDTO cultures of an acinar growth pattern and also from normal (non-malignant) lung organoids. Characteristics of more differentiated LUSC include keratinization and the prominent keratin pearls, and tumors are often very heterogenous. However, in the instances of poorly differentiated LUSC, distinguishing histological features from those of poorly differentiated LUAD can pose a significant challenge, and evaluation of mucus production by AB-PAS staining and subtype-specific markers by IHC-P may be necessary for confirmation.

#### 3.3.1. L4

**Figure 3:**
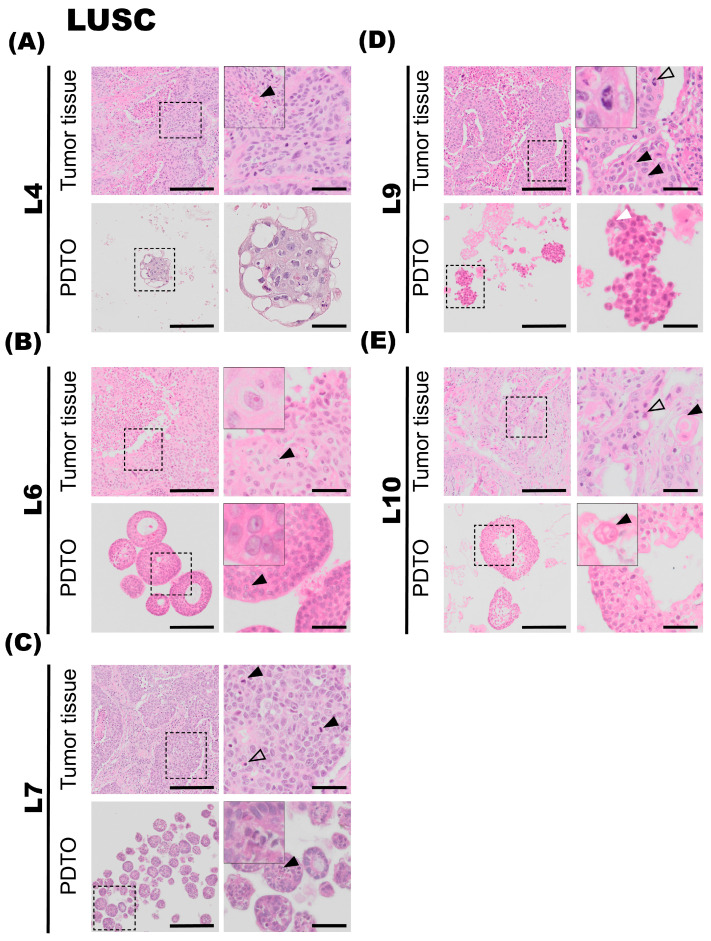
Histology of lung squamous cell carcinoma (LUSC) tumor specimens and the corresponding patient-derived tumor organoid (PDTO) cultures. Histology of LUSC tumor specimens and the corresponding PDTO cultures were assessed on hematoxylin and eosin (H&E)-stained formalin-fixed and paraffin-embedded (FFPE) sections. (**A**) Tumor specimen L4 (upper panel) is a large LUSC, showing moderate-to-large amounts of eosinophilic cytoplasm. Areas of keratinization are observed in a different cell group, indicated with a black arrowhead. L4 PDTO culture (lower panel) grows as compact structures with large and irregularly shaped nuclei. (**B**) Tumor specimen L6 (upper panel) is a LUSC with necrotic foci and a high degree of nuclear pleomorphism. Prominent nucleoli are indicated with a black arrowhead. Prominent nucleoli are also observed in L6 PDTO culture (lower panel, black arrowhead). (**C**) Tumor specimen L7 (upper panel) is a LUSC characterized by moderate-to-sparse cytoplasm and large pleomorphic nuclei, with focal necrotic areas. Numerous mitotic figures are seen (black arrowheads) in addition to apoptotic bodies (transparent arrowhead). L7 PDTO culture (lower panel) consists of compact structures with irregularly shaped cells and nuclei. Mitotic figures are also observed in L7 PDTO (black arrowhead). (**D**) Tumor specimen L9 (upper panel) is a LUSC composed of islands and sheets of epithelial tumor cells with hyperchromatic and pleomorphic nuclei. Intercellular bridges are visible in the tumor specimen (black arrowheads). A mitotic figure is highlighted with a transparent arrowhead. The nuclei of the compact L9 PDTO culture (lower panel) are pleomorphic, and the presence of nucleoli is indicated with a white arrowhead. (**E**) Tumor specimen L10 (upper panel) is a LUSC with irregular chromatin and prominent necrotic areas. The black arrowhead points at an area of keratinization. L10 PDTO culture (lower panel) also consists of areas of keratinization, seen in a different cell group (black arrowhead). Scale bars equal 200 μm (left) and 50 μm (right, zoomed inserts).

The fourth patient specimen (L4) is a large (>7 cm) LUSC. The tumor has areas of prominent necrosis. The epithelial tumor cells grow in cohesive sheets and islands, showing moderate-to-large amounts of eosinophilic cytoplasm, focally with keratinization (Figure 3A, upper panel, black arrowhead). The nuclei are irregular in size and chromatin structure, as seen in Figure 3A. Cytokeratin 5/6 (K5/6) and p40 immunohistochemistry confirmed the LUSC subtype. PD-L1 IHC (clone 22C3) showed a TPS of 20%. Organoids derived from specimen L4 display very similar histology and growth patterns to the corresponding tumor specimen. The malignant cells have large and irregularly shaped nuclei and show signs of keratinization. Thus, there is a high degree of similarity in the characteristics of the organoids and the tumor specimen. Derived organoids from L4 are growing as very compact structures with no vacuole formation. The organoids are typically 100–200 μm in diameter. Further, the AB-PAS stain of organoids shows little-to-no mucin production.

#### 3.3.2. L6

The sixth tumor specimen (L6) has a growth pattern characteristic of LUSC, with epithelial tumor cells arranged in large clusters and sheets, and also shows necrotic foci (Figure 3B). Further, a high degree of nuclear pleomorphism is the observed chromatin structure with prominent nucleoli is also observed (Figure 3B, upper panel, black arrowhead). The epithelial tumor cells are rich in light eosinophilic cytoplasm. Apoptotic bodies and mitotic figures are readily observed. Finally, single-cell keratinization, dyskeratotic cells, some keratin pearls, and intercellular bridges, characteristic of LUSC, were also observed in the specimen. PD-L1 IHC (clone 22C3) showed a TPS of 100%. The organoids derived from tumor specimen L6 display largely similar histological characteristics compared to the corresponding tumor specimen, with a compact growth pattern. Organoid size varies between 50 and 200 μm in diameter and consists of large and irregularly sized cells and pleomorphic nuclei, similar to the malignant tissue they derive from. However, cytoplasmic borders are not sharply demarcated, which may be due to suboptimal preparation of the specimen. Prominent nucleoli are also observed in organoid specimens (Figure 3B, lower panel, black arrowhead). AB-PAS histochemical stain performed on organoids showed no to very little production of mucin.

#### 3.3.3. L7

Tumor L7 is a LUSC with focal necrotic areas (Figure 3C, upper panel). Epithelial tumor cells are characterized by moderate-to-sparse basophilic cytoplasm and large pleomorphic nuclei with condensed chromatin and prominent nucleoli. Some tumor islands show peripheral palisading nuclei. Numerous mitotic figures (Figure 3C, upper panel, black arrowheads) and apoptotic bodies (Figure 3C, upper panel, transparent arrowhead) are seen. Immunohistochemical staining revealed that the tumor was negative for thyroid transcription factor-1 (TTF1), Napsin A. The tumor was focally positive for CK5/6, but not for p40. The neuroendocrine markers chromogranin A and Synaptophysin were both negative. The tumor was partially positive for CD56, a glycoprotein with a role in cell–cell adhesion. This is a more unspecific marker of neuroendocrine origin. Its morphology and immunohistochemical profile are consistent with a poorly differentiated LUSC. PD-L1 IHC (clone SP263) was negative. Ki67 was positive in approximately 50% of the tumor cells. The organoids derived from tumor specimen L7 mostly consist of compact structures and display histological characteristics comparable to the tumor specimen. Furthermore, the organoids consist of irregularly shaped cells and nuclei and have an average diameter of 50–100 μm. Mitotic figures are also observed in L7 PDTO culture (Figure 3C, lower panel, black arrowhead).

#### 3.3.4. L9

The tumor is composed of islands and sheets of epithelial tumor cells with intermediate amounts of eosinophilic cytoplasm. Focal areas containing single-cell keratinization are observed, and intercellular bridges (Figure 3D, upper panel, black arrowhead) are clearly visible in the specimen. Nuclei are hyperchromatic and pleomorphic, some with the presence of distinct nucleoli. Mitotic figures are readily visible (Figure 3D, upper panel, transparent arrowhead). Small areas of necrosis centrally in the tumor islands are observed. The histopathology is consistent with squamous cell carcinoma. PD-L1 IHC (clone SP263) showed a TPS of 80%. The organoids derived from tumor L9 consist of compact structures. Comparable to the tumor specimen, nuclei are pleomorphic, and prominent nucleoli are observed (Figure 3D, lower panel, white arrowhead).

#### 3.3.5. L10

Tumor is characterized by solid islands of medium-to-large epithelial cells with moderate-to-rich eosinophilic cytoplasm. The nuclei are characterized by irregular chromatin, and some of the nuclei are hyperchromatic. Intercellular bridges are seen, and no glandular structures are observed. Areas of keratinization were detected (Figure 3E, upper panel, black arrowhead), as were prominent necrotic areas. Histopathology is consistent with a moderately differentiated squamous cell carcinoma (keratinizing). PD-L1 IHC (clone SP263) was negative. The organoids derived from tumor specimen L10 grew as a mix of compact and hollow organoids. Comparable to the tumor specimen, areas of keratinization are also detected in the organoids, seen in a different cell group (Figure 3E, lower panel, black arrowhead). The organoids were typically 100–200 μm in diameter.

### 3.4. Organoid Cultures Preserve the Protein Expression Pattern and Functional Capabilities of the Respective Patient Tumors in Situ

**Figure 4:**
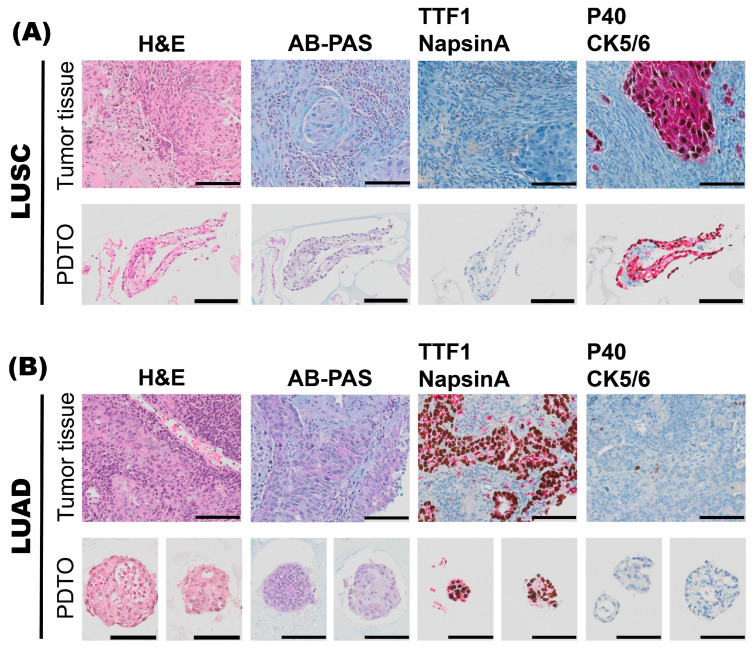
Characterization of histoarchitecture, mucus production, and marker expression of tumor specimens and the corresponding patient-derived tumor organoid (PDTO) culture specimens. A representative case of lung squamous cell carcinoma (LUSC), L10 (**A**), and a representative case of lung adenocarcinoma (LUAD), L11 (**B**), pre-operative biopsies are shown. For both L10 and L11 specimens, histology of archival tumor tissue and PDTO were assessed on hematoxylin and eosin (H&E)-stained sections. Mucus production was assessed by alcian blue–periodic acid–Schiff (AB-PAS) histochemistry. Subtype-specific marker expression was assessed by immunohistochemistry (IHC-P). Dual IHC-P staining for LUAD markers TTF-1 (brown)/Napsin A (red), and LUSC markers p40 (brown)/CK5/6 (red) are shown. Scale bars equal 100 μm.

In cases where it was challenging to determine whether the tumor could be classified as a poorly differentiated LUAD or a poorly differentiated LUSC based on evaluation of histology alone, sections from the pre-operative biopsy or resection specimens were stained with AB-PAS histochemistry to evaluate mucus production. They were also stained with two dual immunohistochemistry (IHC-P) stains: one dual IHC-P stain for LUAD-enriched markers, TTF1 and Napsin A; and one dual IHC-P stain for LUSC-enriched markers, p40 and CK5/6 (Figure 4). A representative case of lung squamous cell carcinoma (LUSC, Figure 4A) and lung adenocarcinoma (LUAD, Figure 4B) is shown.

### 3.5. Molecular Pathological Evaluation of LUADs and Preservation of Genetic Aberrations of the Tumor in the PDTO Cultures

All but one of the adenocarcinomas underwent a complete molecular pathological evaluation to determine potential molecular targets. The molecular pathological analysis included ALK expression evaluated by IHC-P; ROS by IHC-P with confirmation by FISH or NGS; and EGFR, PIK3CA, BRAF, NRAS, and KRAS mutation analyses by NGS, as described. Targetable molecular alterations were only detected in three out of eleven LUAD cases. For L3, the following variant of clinical significance was detected: EGFR exon 19 deletion (Glu746_ala750del). The EGFR exon 19 deletion was confirmed in the organoid tissue by an EGFR Chr 19 del-specific antibody by immunofluorescent (IF) staining on paraffin sections. KRAS G12C mutations were detected in the clinical LUAD specimens L8 (KRAS G12C, nucleotide change c.34G>T) and L11 (KRAS G12C, nucleotide change c.34G>T) by Ampliseq Focus Panel (Illumina) using the Next-Generation Sequencing instrument Miniseq (Illumina) on DNA and RNA isolated from FFPE specimens. When KRAS G12C was not detected in DNA isolated from organoid cultures L8 and L11 by Sanger Sequencing requiring 25–30% tumor cells, a real-time PCR assay requiring only 10% tumor cells was applied: The Thyroid Cancer Mutation Detection Kit (EntroGen) on ABI Applied Biosystems Fast 7500 Real-Time PCR system (ThermoFisher). In L8, no KRAS variant of clinical significance was detected, and in L11, G12C/c.34G>T was detected—however, outside the reference range used for the clinical samples. From these analyses, we conclude that the normal bronchiolar tissue had outgrown the tumor tissue in the organoid cultures prior to DNA isolation, which explains the loss of cancer cells harboring the mutations detected in the tissues of origin.

## 4. Discussion

We aimed to establish an organoid model from human NSCLC resection specimens and perform a thorough characterization of the histoarchitecture and growth pattern of the PDTO cultures compared to the respective tumor specimen they derive from. The establishment of PDTO cultures was performed in two subtypes of NSCLC, namely LUSC and LUAD.

In the process of establishing tumor organoids, both alveolar and bronchiolar differentiation media proved effective for establishing organoids; however, enhanced growth was observed in organoid cultures of the bronchiolar medium. Here, we demonstrate that we, by this procedure, were able to establish PDTO cultures in 11 of 11 NSCLC cases, a success rate of 100%. However, there were variations in the growth kinetics of the PDTO cultures and the individual cultures’ capacity to maintain a pure tumor culture over time. A recent study has shown that the extensive culture ability of lung tumor organoids (passaged > 10 times) correlated with poor patient prognosis [38], and additional growth metric associations for the various subtypes and tumor characteristics would be of interest for future studies. Establishing pure tumor organoids and maintaining a stable success rate remains a significant challenge for extension of the NSCLC PDTO application into the area of use in personalized medicine [29]. Several research groups are actively working on improving the culture medium formulations to avoid the extensive overgrowth of cultures by normal lung organoids. Selective small molecular inhibitors like Nutlin-3a or Palbociclib have been suggested for enriching, respectively, TP53- and RB1-mutated cancer cell-derived organoids at the expense of organoids derived from TP53 and RB1 wildtype cell-derived organoids. Nutlin-3a is an inhibitor of mouse double minute 2 homolog (MDM2), and loss-of-function mutations in TP53 are associated with resistance to Nutlin-3a [39]. Palbociclib, a selective inhibitor of the cyclin-dependent kinases CDK4 and CDK6, was found to enrich tumor organoids with RB1 mutations [40].

Importantly, we found that, in general, PDTO cultures resembled the NSCLC tumor they derived from, with respect to histoarchitecture and growth characteristics. IF antibody stain proved that derived organoids preserved genetic mutation from tumor of origin. We found that the relative difference in mucin production, as determined by AB-PAS histochemistry, was well suited to distinguish poorly differentiated LUAD from LUSC also in the PDTO culture specimens. LUAD-derived PDTO cultures displayed higher mucin production compared to LUSC-derived PDTO cultures, and the scattered mucin expression determined in some of the LUSC specimens likely indicated the presence of patient-derived organoids derived from the expansion of ‘normal’ (non-malignant cell-derived) bronchial cells. We conclude that establishing a close collaboration between research scientists and the clinical departments is a prerequisite for efficient PDTO generation, and the close collaboration with pathologists for excision of tumor material from the freshly resected tumors, as described here, is a critical factor for success and necessary to avoid any potential negative impact on diagnostics. We suggest that monitoring mucus production by AB-PAS staining of the organoid cultures during passaging could be used as a marker to assess the purity of the PDTO culture and to monitor the presence and expansion of ‘normal’ bronchial cell-derived organoids.

We experienced that while most of the organoid cultures could be easily passaged over time, some cultures were challenging to propagate in culture. With propagation and passaging of the culture, the tumor cells were lost with time. For example, L8 organoids, which were derived from a LUAD with a papillary growth pattern, were difficult to establish. For L11 organoid culture, which is a LUAD of mixed growth pattern with both differentiated (papillary) and less differentiated (solid) areas, we experienced that normal lung organoids expanded well compared to the cancer cell organoids. Still, the normal organoids took over the culture with time, which is reflected in the fact that we were not successful in validating the KRAS G12C variant in the cultured organoids. A tendency for LUADs of papillary and lepidic growth patterns to have a better prognosis compared to LUADs displaying an acinar or solid growth pattern has been shown [41,42]. Our study supports the work of others, showing that the success rate of PDTO culture in highly differentiated NSCLC may be lower than in poorly differentiated NSCLC [43]. Although no direct correlation exists between growth patterns and the stage of the cancer it is derived from, the establishment and growth rates of lung tumor organoids have been shown to be associated with histological subtype and tumor size. In addition, in other studies, the lepidic-predominant LUADs were particularly challenging to establish and maintain in culture [38]. Of note, L8 and L11 also harbor KRAS G12C mutations. A larger dataset and quantitative growth metrics would be required to conclude whether the ability to form organoids in culture and to sustain the malignant organoid culture growth for prolonged periods of time is linked to the growth pattern of the tumors they derive from.

For LUADs, the frequent occurrence of molecular aberrations (e.g., EGFR and ALK) provides opportunities for targeted therapies. However, resistance to targeted monotherapies inevitably develops, and optimal treatment selection following standard-of-care treatment algorithms is often unclear [44]. Lung tumor organoid cultures harboring clinically actionable genetic variants have been shown to mirror the response of the tumor they derive from with respect to targeted therapies [38]. The PDTO model offers an alternative to guide treatment selection in this context [29] and a promising research tool to study mechanisms of therapy resistance.

In another study, Djikstra and colleagues showed that a majority of organoids derived from intrapulmonary lung cancers were overgrown by normal airway organoids, and that the overall establishment rate of pure lung cancer organoids was only 17% [29]. These numbers restrict the utility of lung cancer organoids in personalized medicine approaches and call for improved culture protocols to establish pure cultures, and careful monitoring of culture purity. Copy number profiling and immunostaining may be used to distinguish PDTO from normal PDO in culture. In line with the findings of others [29], we conclude that reliable classification of lung organoid cultures derived from intrapulmonary tumor tissue is essential, and close collaboration with thoracic pathologists is needed to firmly perform the classification based on histopathology and additional marker expression patterns. We believe that the thorough histopathological evaluation of PDTOs in this study will serve as a useful framework for researchers aiming to implement PDTO cultures in their translational research projects and inspire the development and integration of PDTO models in translational research projects. While EGFR and KRAS variants were evaluated in this study, broader multi-omics profiling, including copy number variation and transcriptomics, could be applied in future studies for surveillance of organoid cultures and to strengthen the claim of organoid fidelity to parental tumors [27,38].

Testing the effectiveness of chemotherapeutics or targeted therapies in the established model was not the main aim of this study. However, drug sensitivity testing in lung tumor organoid cultures has been shown to successfully predict clinical response to targeted agents in NSCLC cases where rare mutations of unknown clinical significance in targetable genes were detected [38], and future studies will serve to determine the extent to which utilization of PDTO cultures can aid in treatment selection and the development of novel treatment strategies in cases where genetic variants of unknown clinical significance are detected. Furthermore, future studies would benefit from the careful monitoring of how well potentially targetable gene variants are preserved in the PDTO cultures. As lung cancer remains a leading cause of cancer-related morbidity and mortality worldwide, this approach to target genetic alterations affecting only a minority of patients could still potentially benefit the lives of many patients. In order to optimize clinical benefit, it would be useful to explore in future studies whether smaller tissue samples from either transthoracic fine-needle aspiration (FNA) samples, endo-bronchial ultrasound-guided transbronchial needle aspiration (EBUS-TBNA), and biopsies (transbronchial or transthoracic) may be used to establish organoid cultures. Additionally, determining whether these samples could be beneficial for functional drug testing aimed at achieving personalized lung cancer treatment is essential.

## 5. Conclusions

Organoid models are emerging as a translational bridge between basic biomedical science and clinical medicine. We have successfully established a multicellular 3D model of patient-derived tumor organoids (PDTOs) from non-small-cell lung cancer (NSCLC) tissues, and we have shown, through robust tissue-based analyses, that the histopathological features of the organoid cultures resemble the respective tissues they derive from. The biomimetic organoid models hold great potential to increase our understanding of the molecular mechanisms underpinning disease pathophysiology and therapy resistance, and tumor organoid models also hold remarkable promise for personalized medicine as patient-specific models, and even to predict therapy response in cases where molecular–pathological analyses pose significant management dilemmas. The lack of a thorough histopathological analysis has limited the acceptance of organoid models as translational tools, and the realization of their full potential. We believe the comprehensive characterization of PDTO culture and histopathological characterization described here will be of value for future studies seeking to apply these biologically relevant tools in translational research projects and co-clinical trials.

## Figures and Tables

**Table 1:** Organoid washing media (AdDF+++).

Media Component	Supplier	Catalogue Number	Final Concentration
Advanced DMEM/F12	Invitrogen (Waltham, MA, USA)	12634-034	1×
Glutamax	Invitrogen (Waltham, MA, USA)	12634-034	1×
HEPES	Invitrogen (Waltham, MA, USA)	15630-056	10 mM
Penicillin/streptomycin	Invitrogen (Waltham, MA, USA)	15140-122	1×

**Table 2:** Organoid culture media.

Media Component	Supplier	Catalogue Number	Final Concentration
R-Spondin-1	Peprotech, Cranbury, NJ, USA	120-38	500 ng/mL
FGF-7	Peprotech, Cranbury, NJ, USA	100-19	25 ng/mL
FGF-10	Peprotech, Cranbury, NJ, USA	100-26	100 ng/mL
Noggin	Peprotech, Cranbury, NJ, USA	120-10C	100 ng/mL
A83-01	Tocris, Bristol, UK	2939	500 nM
SB202190	Sigma-Aldrich, St. Louis, MO, USA	S7067	500 mM
Y-27632 ^1^	StemCell technologies, Cambridge, MA, USA	72304	5 mM
CHIR99021 ^2^	StemCell technologies, Cambridge, MA, USA	72054	3 μM
N-acetylcysteine	Sigma-Aldrich, St. Louis, MO, USA	A9165-5g	1.25 mM
Nicotinamide	Sigma-Aldrich, St. Louis, MO, USA	N0636	5 mM
Glutamax	Invitrogen, Waltham, MA, USA	12634-034	1×
HEPES	Invitrogen, Waltham, MA, USA	15630-056	10 mM
B-27	Gibco, Taastrup, Denmark	17504-44	1×
Penicillin/streptomycin	Invitrogen, (Waltham, MA, USA)	15140-122	100 U·mL^−1^/100 mg·mL^−1^
Advanced DMEM/F12	Invitrogen, (Waltham, MA, USA)	12634-034	1×

^1^ Y-27632 was only added the first 4 days of culture. ^2^ CHIR99021 was added in AO-O medium to promote alveolar differentiation (final conc. 3 μM).

**Table 3:** The characteristics of NSCLC subtype and pTNM classification of specimens included in this study.

Sample ID	Gender	NSCLC Subtype	pTNM Classification (TNM 8th Ed.)
L1	M	LUAD	pT1b N0 M0
L2	F	LUAD	pT1c N0 M0
L3	F	LUAD	pT2a N0 M0
L4	M	LUSC	pT4 N2 M0
L5	M	LUAD	T4 N0 M0
L6	M	LUSC	pT3 N0 M0
L7	M	LUSC	pT2a N0 M0
L8	M	LUAD	pT2b N0 M0
L9	F	LUSC	pT2b N0 M0
L10	M	LUSC	pT2b N0 M0
L11	M	LUAD	pT2a N0 M0

## Data Availability

The original contributions presented in this study are included in the article. Further inquiries can be directed toward the corresponding authors.
